# Morphoproteomic Profiling of the Mammalian Target of Rapamycin (mTOR) Signaling Pathway in Desmoplastic Small Round Cell Tumor (EWS/WT1), Ewing’s Sarcoma (EWS/FLI1) and Wilms’ Tumor(WT1)

**DOI:** 10.1371/journal.pone.0068985

**Published:** 2013-07-29

**Authors:** Vivek Subbiah, Robert E. Brown, Yunyun Jiang, Jamie Buryanek, Andrea Hayes-Jordan, Razelle Kurzrock, Pete M. Anderson

**Affiliations:** 1 Department of Investigational Cancer Therapeutics, Division of Cancer Medicine, The University of Texas MD Anderson Cancer Center, Houston, Texas, United States of America; 2 Department of Pathology and Laboratory Medicine, University of Texas Health Science Center-Medical School at Houston, Houston, Texas, United States of America; 3 Department of Surgical Oncology, The University of Texas MD Anderson Cancer Center, Houston, Texas, United States of America; 4 Center for Personalized Therapy & Clinical Trials Office, Division of Hematology & Oncology, University of California San Diego - Moores Cancer Center, La Jolla, California, United States of America; 5 Pediatric Hematology/Oncology/BMT, Levine Children’s Hospital/Levine Cancer Institute, Charlotte, North Carolina, United States of America; Tulane University School of Medicine, United States of America

## Abstract

**Background:**

Desmoplastic small round cell tumor (DSRCT) is a rare sarcoma in adolescents and young adults. The hallmark of this disease is a EWS-WT1 translocation resulting from apposition of the Ewing’s sarcoma (*EWS*) gene with the Wilms’ tumor (*WT1*) gene. We performed morphoproteomic profiling of DSRCT (EWS-WT1), Ewing’s sarcoma (EWS-FLI1) and Wilms’ tumor (WT1) to better understand the signaling pathways for selecting future targeted therapies.

**Methodology:**

This pilot study assessed patients with DSRCT, Wilms’ tumor and Ewing’s sarcoma. Morphoproteomics and immunohistochemical probes were applied to detect: p-mTOR (Ser2448); p-Akt (Ser473); p-ERK1/2 (Thr202/Tyr204); p-STAT3 (Tyr 705); and cell cycle-related analytes along with their negative controls.

**Principal Findings:**

In DSRCT the PI3K/Akt/mTOR pathway is constitutively activated by p-Akt (Ser 473) expression in the nuclear compartment of the tumor cells and p-mTOR phosphorylated on Ser 2448, suggesting mTORC2 (rictor+mTOR) as the dominant form. Ewing’s sarcoma had upregulated p-Akt and p-mTOR, predominantly mTORC2. In Wilm’s tumor, the mTOR pathway is also activated with most tumor cells moderately expressing p-mTOR (Ser 2448) in plasmalemmal and cytoplasmic compartments. This coincides with the constitutive activation of one of the downstream effectors of the mTORC1 signaling pathway, namely p-p70S6K (Thr 389). There was constitutive activation of the Ras/Raf/ERK pathway p-ERK 1/2 (Thr202/Tyr204) expression in the Wilms tumor and metastatic Ewing’s sarcoma, but not in the DSRCT.

**Conclusion:**

Morphoproteomic tumor analyses revealed constitutive activation of the mTOR pathway as evidenced by: (a) expression of phosphorylated (p)-mTOR, p-p70S6K; (b) mTORC 2 in EWS and DSRCT; (c) ERK signaling was seen in the advanced setting indicating these as resistance pathways to IGF1R related therapies. This is the first morphoproteomic study of such pathways in these rare malignancies and may have potential therapeutic implications. Further study using morphoproteomic assessments of these tumors are warranted.

## Introduction

Desmoplastic small round cell tumor (DSRCT) is a rare sarcoma that presents as diffuse peritoneal sarcomatosis in children, adolescents and young adults, predominantly males [1], [2]. Because of its rarity, it is often misclassified as being other small round blue cell tumors. Ewing’s sarcoma (ES) is the second most common bone tumor in children, adolescents and young adults. Traditionally, DSRCT patients have been treated similarly to ES patients using an algorithm that incorporates surgery, radiation therapy, and multidrug chemotherapy [3]. The prognosis of relapsed DSRCT is poor and achievement of durable remission remains elusive [2].

Wilms’ tumor (WT), or nephroblastoma, is a neoplasm of the kidneys that typically occurs in children [4]. The prognosis is dependent on histology and staging [5]. The use of chemotherapy combined with surgery and radiotherapy has greatly improved the outcome of WT patients, with more than 90% being cured [6]. However, the treatment of advanced and high-risk WT remains a challenge.

DSRCT, WT and ES share a chimeric relationship with one another. DSRCT is caused by the translocation of the *EWSR1* gene from chromosome 22 to chromosome 11, resulting in a fusion product EWSR1/WT1 [7]. ES is caused by the translocation of the EWSR1 gene from chromosome 22 to chromosome 11 in most cases (EWSR1-FLI1) and chromosomes 21 [8] and 7 in rare cases (EWSR1-ERG and EWSR1-ETV1) [9], [10]. Mutation of the WT1 gene on chromosome 11 is observed in 20% of WT cases [11]. A CTNNB1 mutation is also seen in 14% of WT cases and the WT1 and CTNNB1 mutations are highly associated [12]. Because DSRCT, ES and WT seem to be genetically close cousins, presenting in young patients and responding to similar cytotoxic active agents, comparing the signaling pathways is a rational approach toward delineating the biology of these rare tumors.

Here, we report our experience with two index cases of DSRCT and WT with a detailed clinical history and assessment of morphoproteomic signaling for profiling and comparing two previously reported ES cases to elucidate current and future therapeutic options [13]. We show mTOR pathway signaling in these interesting tumors. Moreover, ERK pathway signaling seen in the advanced setting suggests that it is a resistance pathway.

## Methods

### Patient Selection

We reviewed the medical records of a patient with DSRCT and a patient with WT. Both patients were seen in the Departments of Investigational Cancer Therapeutics and Pediatrics at The University of Texas MD Anderson Cancer Center (MD Anderson). This study was performed in accordance with the guidelines of the MD Anderson Institutional Review Board (IRB), as previously described [13].Because this was a retrospective chart review IRB has waived the consent requirements.

### Immunohistochemical and Morphoproteomic Analyses

Immunohistochemical and morphoproteomic analyses of tumor samples collected from each patient were performed, as previously described [13]. Briefly, this pilot study assessed two patients, one with DSRCT (Pt #1), and the other with Wilms’ tumor (#2) and compared them with our previously reported ES patients. Morphoproteomics and immunohistochemical probes [13] were used to detect p-mTOR (Ser2448), p-Akt (Ser473), p-ERK1/2 (Thr202/Tyr204), p-STAT3 (Tyr 705), VEGF-A expression, cell cycle related analytes including Ki67, cyclin-D1 and Skp 2 along with negative and positive controls. All analyses were performed at The University of Texas at Houston certified under the Clinical Laboratory Improvement Amendments of 1988 (“CLIA”) as qualified to perform high-complexity clinical testing.

## Results

The clinical history of the four patients in this pilot studies and their responses to chemotherapy and targeted therapy are reviewed below.

### 

#### Patient 1

A sixteen-year-old white male presented with massive abdominal distension and dyspnea that worsened with exertion. A PET/CT scan revealed extensive abdominal disease, including a dominant 28 cm mass, all of which were FDG avid. A biopsy showed a small round blue cell tumor consistent with the diagnosis of DSRCT. The patient was initially treated with chemotherapy, similar to the treatment of ES, consisting of vincristine, adriamycin, and cyclophosphamide, alternating with etoposide and ifosfamide for a total for 3 cycles. He then received sunitinib(oral small molecule, multi-targeted anti-angiogenic receptor tyrosine kinase inhibitor) for 6 weeks and was referred to MD Anderson for further management. The patient received 5 cycles of systemic therapy before harvesting his stem cells. He then underwent cytoreductive surgery followed by hyperthermic intraperitoneal cisplatin. His next treatment regimen was temozolomide and irinotecan before whole abdominal radiation therapy, followed by switching to weekly vinorelbine with daily oral cyclophosphamide. Following relapse, the patient was started on pazopanib, a multi-kinase inhibitor anti-angiogenic agent, and the mTOR inhibitor rapamycin before switching again to vincristine, adriamycin, cyclophosphamide alternating with etoposide and ifosfamide for a total for 6 cycles. He was then given temozolomide concomitant with radiation before eventually receiving PEG-interferon at the time of last follow up.

#### Patient 2

A sixteen-year-old white female was initially diagnosed with Wilms’ tumor by radical nephrectomy. The patient received actinomycin-D and vincristine and developed recurrence in the lungs. Biopsy of the lung nodule confirmed Wilms’ tumor. She received 1 cycle of vincristine plus doxorubicin before switching to cyclophosphamide plus etoposide. She then received “ICE-T” (ifosfamide, etoposide, carboplatin plus topotecan) before undergoing a double autologous stem cell rescue with cyclophosphamide, carboplatin, etoposide and melphalan. This was followed by radiation to the lungs. After developing progression in lung metastasis, she was enrolled on a phase I study with sorafenib(multi-kinase anti-angiogenic inhibitor targeting several tyrosine protein kinases including the Raf kinases ), resulting in a 25% decrease in the size of her lung metastases followed by stable disease for 8 months. After progression, she was enrolled on a phase I study of temsirolimus, a mammalian target of rapamycin (mTOR) inhibitor, in combination with the insulin-like growth factor (IGF1R) inhibitor IMC-A12. Unfortunately she progressed on this treatment. She was then treated with rapamycin and sunitinib, with discontinuation of rapamycin following grade 4 thrombocytopenia. She then progressed on liposomal doxorubicin+bevacizumab (avastin) and then liposomal doxorubicin+bortezomib (velcade, proteasome inhibitor). She was then referred to the Phase I Clinic at MDAnderson, enrolled on a checkpoint kinase (CHK) inhibitor study, and then, unfortunately, she progressed on therapy and was taken off the study.

#### Patients 3 & 4 (ES)

Briefly, patients #3 and #4 were a 24-year-old white female and 21-year-old white male diagnosed with ES. Both patients progressed on several lines of standard chemotherapy for ES and were referred for enrollment on Phase I clinical trials. Interestingly, both patients had a significant response to single agent Insulin like growth factor 1 receptor (IGF1R) antibodies. Following progression on single-agent IGF1R antibody treatment, both patients were enrolled on a combined IGF1R+mTOR inhibitor therapy. Patient #3 continues on therapy for more than 6 years on IGF1R inhibitor based therapy, whereas patient #4 progressed. The results with detailed correlative morphoproteomics have been published [13].

### Morphoproteomics/Immunohistohemistry and Correlative Studies

**Figure 1:**
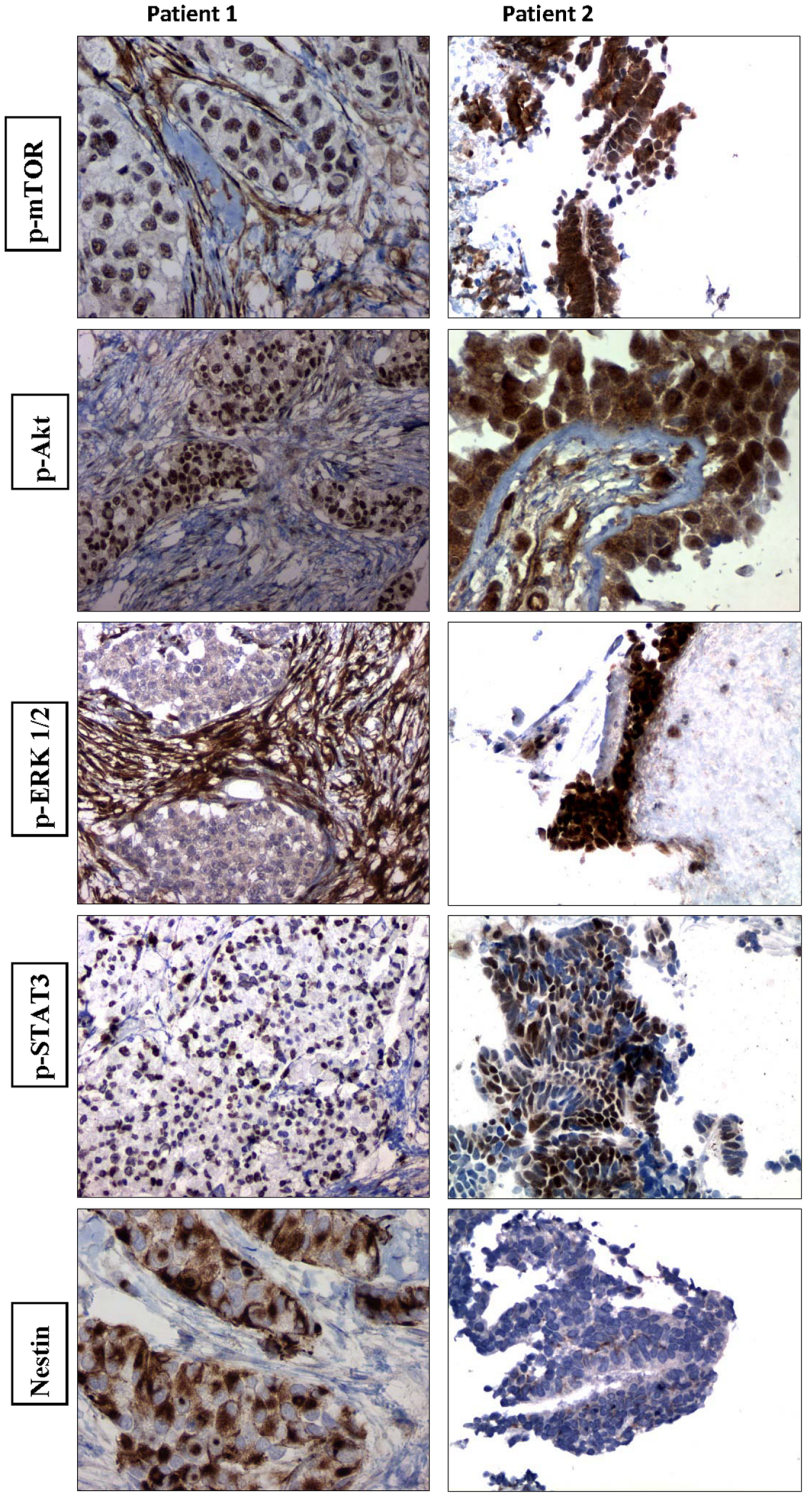
Morphoproteomics and Immunohistochemistry Patients #1, 2. In patient #1 (DSRCT) the PI3K/Akt/mammalian target of rapamycin (mTOR) pathway is constitutively activated by p-Akt (Ser 473) expression in the nuclear compartment of the tumor cells and p-mTOR phosphorylated on Ser 2448, suggesting mTORC2 (rictor+mTOR) as the dominant form. In patient #2 (Wilm’s tumor), the mTOR pathway is also activated with most tumor cells moderately expressing p-mTOR (Ser 2448) in plasmalemmal and cytoplasmic compartments. This coincides with the constitutive activation of one of the downstream effectors of the mTORC1 signaling pathway, namely p-p70S6K (Thr 389). There is constitutive activation of the Ras/Raf kinase/extracellular signal-regulated kinase (ERK) pathway p-ERK 1/2 (Thr202/Tyr204) expression in the Wilms tumor.

The morphoproteomics results are summarized in Table 1 and Figure 1.

**Table 1:** Morphoproteomic profiling of patients with DSRCT, Wilms’ tumor and Ewing’s sarcoma.

Patient # and Diagnosis*	P #1 DSRCT	Pt #2 Wilms’Tumor	Pt #3 Ewing’s sarcoma		Pt #4 Ewing’s sarcoma	
Biopsy Timeline			Before IGF1R	After IGF1R	Before IGF1R	After IGF1R+mTOR
**p-mTOR** †	1–2+,N, stromalcells 2–3+	2+, P-C, >50%	±−1+/±−1+	1–3+/1+	0–2+/±	1–3+/±
**p-Akt** †	±−3+, N,	1+/1+, N/C, ∼90%	0−±/±−1+	1–3+/1–2+	0–2+/±	±−3+/±
**p-STAT3** †	0–3+, N	2–3+, N, ∼20%			0–3+	0–3+
**Nestin** †	0–3+, C/P, majority	0	0	0	0–1+	0–3+
**p-ERK1/2** † ‡	0−±, N,	3+/2+, N/C, ∼90%	N/A	N/A	0–3+, N/C	1–3+/±

* Scoring intensity graded on a scale of 0 (no signal) to 3+ (high intensity). N-Nuclear,C-Cytoplasmic,P-Plasmalemmal.

† Plasmalemmal;

‡ Thr 202/Tyr 204 [Nuclear].

#### Patient 1 (DSRCT)

Sections of tumor tissue from cytoreductive surgery were available. There is minimal constitutive activation of the Ras/Raf kinase/ERK pathway as evidenced by the expression of phosphorylated (p-) extracellular signal-regulated kinase (ERK) 1/2 (Thr 202/Tyr204) with nuclear translocation in only a rare tumor cell. It may be relevant that vincristine can cause inactivation of ERK [14]. In contrast, the PI3K/Akt/mTOR pathway is constitutively activated by the expression of p-Akt (Ser 473), almost exclusively in the nuclear compartment of the tumor cells and of p-mTOR phosphorylated on Ser 2448, also with variable mild to moderate nuclear expression. This pattern of expression with nuclear subcellular compartmentalization suggests that mTORC2 (rictor+mTOR) is the dominant form [15], [16], [17], [18]. Most of the tumor cells were also immunopositive for nestin, a neural precursor/differentiation marker. Nestin has been identified and reported previously in DSRCT [19]. Notably, a minor but significant portion of the tumor nuclei express p-signal transducer and activator of the transcription (STAT) 3 (Tyr 705) in their nuclei in some regions (Figure 1).

#### Patient 2 (WT)

Lung biopsy was performed after the patient progressed on doxil+bortezomib(velcade). The mTOR pathway is activated, with the vast majority of tumor cells showing moderate expression of p-mTOR (Ser 2448) on the plasmalemmal and cytoplasmic compartments. This coincides with constitutive activation of a downstream effector of the mTORC1 signaling pathway, p-p70S6K (Thr 389) in ∼90 to 95% of tumoral nuclei [20]. The expression of both nuclear and cytoplasmic p-Akt (Ser 473) raises the possibility of mTORC2 pathway signaling as well. There is constitutive activation of the ras/Raf kinase/ERK pathway in the form of p-ERK ½ (Thr202/Tyr204) expression, showing nuclear translocation [21] in ∼90% of the tumor cells. In addition, the STAT pathway appears activated in approximately 20% of the tumor with nuclear translocation of p-STAT3 (Tyr 705). Nestin is negative or weakly expressed.

#### Patients 3 and 4

Briefly, in the two patients with ES, p-Akt and p-mTOR, predominantly mTORC2, were upregulated in initial biopsies before the IGF1R inhibitor therapy. In patient #4, who responded to the combined IGF1R+mTOR targeted therapy and then relapsed, the resistant tissue showed activation of the ERK pathway as well. This was speculated from the evidence of constitutive activation of the p-ERK 1/2 (Thr202/Tyr204) expression, showing nuclear translocation (Table 1).

## Discussion

**Figure 2:**
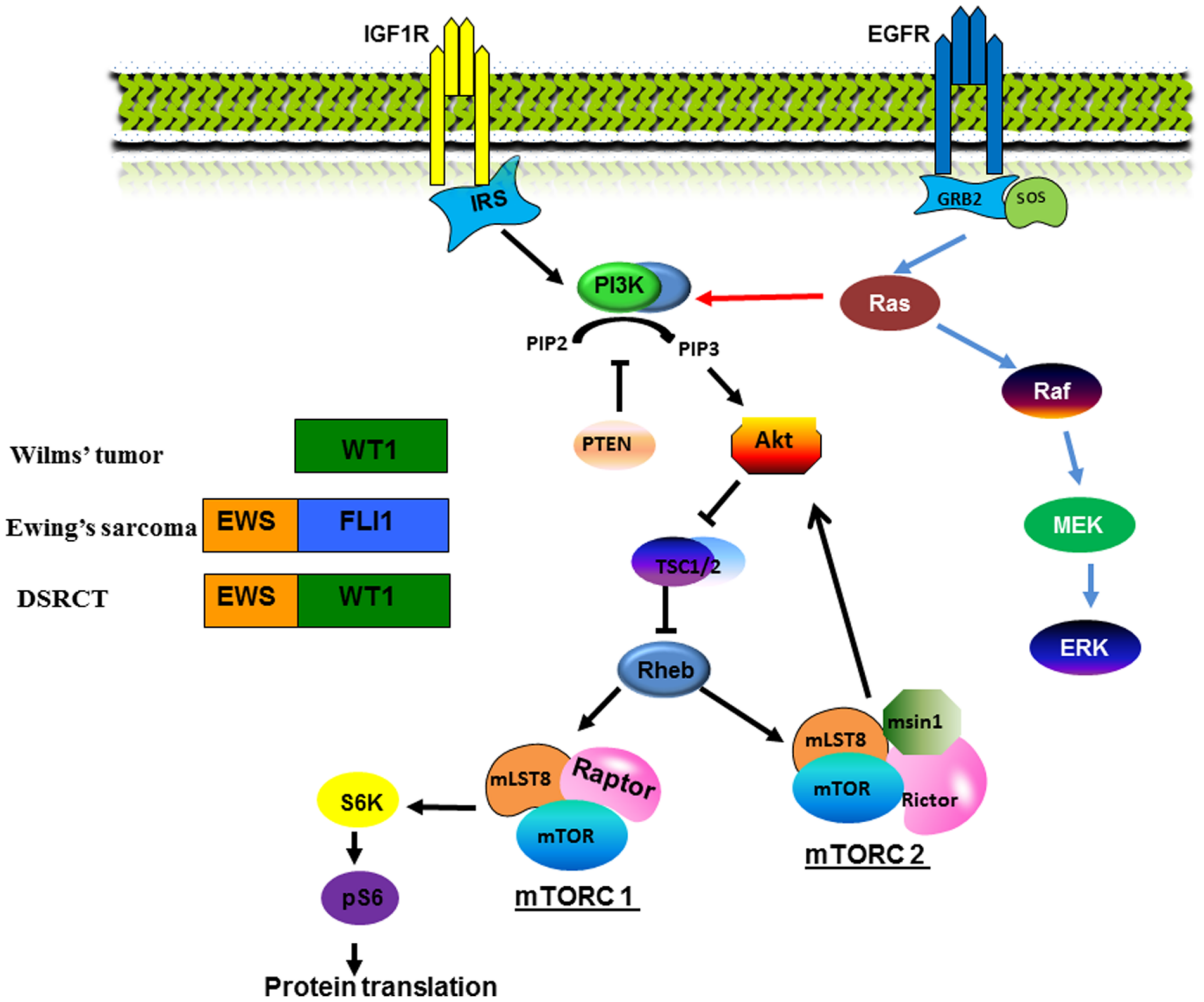
Simplified mTORC 1 and 2 pathway and the relationship with WT1, EWS-FLI-1 and EWS-WT1. Morphoproteomic analyses shows a similarity among DSRCT, ES and WT in the constitutive activation of the mTOR pathway, evidenced by the expression of p-mTOR and p-p70S6K. We also found that mTORC2 is the dominant form in both ES and DSRCT, whereas mTORC1 is activated in WT. Our results also suggest a mechanism that may be used to predict response to IGF1R/mTOR inhibitor therapy, which is to compare Akt/mTOR pathway and Ras/Raf/ERK pathway activation. If the IGF1R/mTOR pathway is the dominant pathway, the patient may respond to IGF1R-related therapy.

We have shown the morphoproteomic signaling pathways of a DSRCT patient and a WT patient and compared them to two previously reported patients with ES. [13] In this small observational study we demonstrated the mTOR signaling pathway involvement among these three interesting tumors(Figure 2). In the patient with DSRCT the PI3K/Akt/mTOR pathway is constitutively activated by virtue of the expression of p-Akt (Ser 473) in the nuclear compartment of the tumor cells, and p-mTOR phosphorylated on Ser 2448, suggesting mTORC2 (rictor+mTOR) as the dominant form. In the patients with ES, there was upregulation of p-Akt and p-mTOR, predominantly mTORC2. In the patient with WT, the mTOR pathway is also activated with most tumor cells showing moderate expression of p-mTOR (Ser 2448) in the plasmalemmal and cytoplasmic compartments. This coincides with the constitutive activation of one of the downstream effectors of the mTORC1 signaling pathway, p-p70S6K (Thr 389). There was constitutive activation of the ERK, with p-ERK 1/2 (Thr202/Tyr204) expression in the WT and ES patients in the advanced setting, but not in the DSRCT patient.

**Figure 3:**
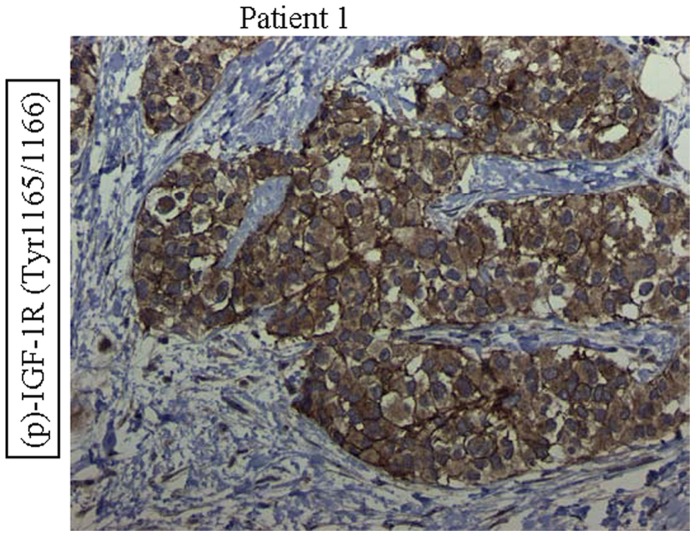
Morphoproteomic analysis of insulin-like growth factor(IGF) pathway. Morphoproteomic analysis of insulin-like growth factor(IGF) pathway reveals constitutive activation of IGF-1receptor as evidenced by the expression of phosphorylated (p)-IGF-1R (Tyr1165/1166) on the plasmalemmal aspect and in cytoplasmic compartments of the tumor cells in DSCRT (Patient #1). Note overexpression vis-à-vis the tumoral stroma (original magnification ×400).

In patient #1 (DSRCT), the deactivation of Ras/Raf/ERK pathway is consistent with a previous finding that vincristine can inactivate ERK [14]. Meanwhile, it could be speculated that activation of p-Akt caused activation of the PI3K/Akt/mTOR pathway, which provided a bypass for the Ras/Raf/ERK pathway and resulted in subsequent tumor growth. The expression pattern of p-mTOR suggested that mTORC2 is the dominant form in DSRCT. Of interest, mTORC2 is also the dominant form in ES, as previously reported. The next rational treatment regimen for patient #1 would be rapamycin, which after long exposure, suppresses the formation of mTORC2, thus inhibiting the mTORC2 pathway [17]. Given this molecular background, the patient was treated with pazopanib and rapamycin combination therapy, but was taken off therapy after 10 days due to a pulmonary embolism. Because mTORC2 phosphorylates S473 of AKT, an AKT inhibitor might be of benefit in the next line of treatment. In addition, IGF1R-related therapy is also a candidate as IGF1R is one of the key regulators of the mTORC2 pathway. Of interest morphoproteomic analysis of insulin-like growth factor(IGF) pathway reveals constitutive activation of IGF-1receptor as evidenced by the expression of phosphorylated (p)-IGF-1R (Tyr1165/1166) on the plasmalemmal aspect and in cytoplasmic compartments of the tumor cells in DSCRT (Patient#1) (Figure 3).

In patient #2 (WT), the constitutive activation of mTORC1 is evidenced by the activation of p-p70S6K (Thr 389). In addition, the expression of both nuclear and cytoplastmic p-Akt (S473) also suggests mTORC2 pathway signaling. The activation of both mTORC1 and mTORC2 could be consistent with IGF1R signaling reported in WT [22]. However, in spite of activated p-Akt and the mTOR pathway, patient #2 did not respond to IGF1R+mTOR inhibitor therapy.

To explicate mechanisms of tumor resistance, we compared patient #2 with two EWS patients (patients 3&4) who initially responded to IGF1R+mTOR inhibitor therapy. In fact, patient #3 remains on therapy, whereas patient #4 developed resistance after four months [13]. Comparing the three patients suggests that the activation of Ras/Raf/ERK pathway may be a key player in diverse responses. The Ras/Raf/ERK pathway provides a bypass for the mTOR pathway and, therefore, may result in tumor resistance. This is consistent with the observation that activation of the Ras/Raf/ERK pathway was observed in the tumor both of patient #2 and the tumor resistance of patient #4. We then compared the p-ERK1/2 level between those two patients and found that the level of p-ERK level expression in patient #2 was significantly higher than in patient #4, while the levels of p-Akt and p-mTOR were comparable between both patients. These results suggest that the Ras/Raf/ERK pathway may be the dominant pathway in patient #2′s tumor. In contrast, patient #4′s tumor was initially regulated primarily by the Akt/mTOR pathway, which explains his initial response to the therapy. However, the Ras/Raf/ERK pathway then gained dominance, driving tumor resistance. Therefore, the next logicial line of treatment for patient #2 would likely be a ERK/MEK inhibitor combined with IGF1R+mTOR inhibitors.

Morphoproteomic analyses revealed a similarity among DSRCT, ES and WT in the constitutive activation of the mTOR pathway, evidenced by the expression of p-mTOR and p-p70S6K. We also found that mTORC2 is the dominant form in both ES and DSRCT, whereas mTORC1 is activated in WT. Our results also suggest a mechanism that may be used to predict response to IGF1R/mTOR inhibitor therapy, which is to compare Akt/mTOR pathway and Ras/Raf/ERK pathway activation. If the IGF1R/mTOR pathway is the dominant pathway, the patient may respond to IGF1R-related therapy. Otherwise the patient’s tumor might be resistant to IGF1R inhibitor therapy. Although we realize these are very preliminary observations on a few patients morphoproteomic analysis provided insights that need to be confirmed in larger samples to avoid sampling error as well as increased numbers of these 3 tumors. If mTOR is confirmed, this may have potential implications for future molecularly targeted therapies to inhibit metastases of these small round blue-cell malignancies. One limitation of the study is that the examined tissues are post-chemotherapy resected tumors and that activation of mTOR pathway could be due to chemotherapy treatment rather than by the tumor-specific genetic or epigenetic alterations.

In summary, morphoproteomic analyses of the patients’ tumors in this pilot study revealed constitutive activation of the mTOR pathway as evidenced by: (a) expression of phosphorylated (p)-mTOR and p-p70S6K; (b) mTORC 2 in ES and DSRCT; (c) ERK signaling was seen in the advanced setting, pointing to these as resistance pathways to IGF1R+mTOR-based therapies. This is the first morphoproteomic study demonstrating mTOR signaling pathway in these rare malignancies and may have potential therapeutic implications. Further morphoproteomic assessments of these tumors are warranted.
